# Health literacy as a buffer: mitigating the impact of Multimorbidity on functional health in older adults

**DOI:** 10.1007/s40520-025-03259-2

**Published:** 2025-12-03

**Authors:** Aline Schönenberg, Tino Prell

**Affiliations:** 1 Department of Geriatrics, Halle University Hospital, Halle (Saale), Germany; 2 Department of Geriatrics, Jena University Hospital, Jena, Germany

**Keywords:** Health literacy, Multimorbidity, Geriatric patients, Older adults, ADLs : activities of daily living

## Abstract

**Background:**

Multimorbidity is a leading cause of functional health impairments in older adults, affecting Activities of Daily Living (ADL). Health-literacy enables individuals to access, process, and apply health-related information effectively, serving as a strategy to mitigate these effects. Aims: This study explores the moderating role of health literacy in the relationship between multimorbidity and functional health according to ADL.

**Methods:**

Data were derived from 3069 individuals aged 80 and older from the “Ageing in Germany (D80+)” survey. Multimorbidity was measured using a 22-item index, while health literacy was assessed via a two-item scale evaluating knowledge and compliance. Functional health was determined by ADL performance. Elastic Net regression and moderation analysis were employed to examine the relationships between multimorbidity, health literacy, and functional health, controlling for sociodemographic and mental covariates.

**Results:**

Multimorbidity was significantly associated with functional health (b= -1.668, *p* < 0.001). Health literacy emerged as a significant moderator, attenuating the impact of multimorbidity on functional health (interaction term: b = 0.243, *p* = 0.023). Conditional effects analysis revealed that individuals with higher health literacy exhibited better functional health, with the adverse effect of multimorbidity on ADL substantially diminished in this group.

**Discussion:**

This study highlights the critical role of health literacy in mitigating the impact of multimorbidity on functional health. Interventions aimed at enhancing health literacy offer a promising avenue for promoting independence and functional health in older adults.

**Conclusion:**

Future research should focus on longitudinal designs and objective measures to further elucidate the pathways linking multimorbidity, health literacy, and functional health. Fostering the ability to independently obtain, understand and implement health information should be a key goal of clinical practice and policy interventions.

## Introduction

Increased life expectancy brings with it an increased risk of age-related health impairments [1]. The aging global population is therefore resulting in a notable increase in the prevalence of multimorbidity and reduced functional health among older adults [2, 3]. The term “multimorbidity” is used to describe the presence of two or more chronic conditions in an individual. While it is estimated that about half of the population aged 60 or above suffers from multimorbidity [2], its prevalence increases along with age [4], with German data showing that people above 80 years of age were on average treated for 3.6 concurring illnesses, with a third even being treated for 5 or more [5]. Another study on German adults showed that in the group aged 60–69, around 62% of people suffered from multimorbidity while this number rose to 78% in those aged 80 or older [6].

This high prevalence is all the more detrimental as multimorbidity has been identified as a significant factor influencing the quality of life and autonomy as well as functional health in older persons [2, 7]. Functional health, frequently assessed through the ability to perform (Instrumental) Activities of Daily Living (ADL), including activities such as bathing, dressing, and eating, serves as a pivotal indicator of independence in this population [8]. Reduced functional health not only impacts personal autonomy but also increases the reliance on caregivers and burden on healthcare systems.

To address the burden of multimorbidity, it is necessary to implement targeted interventions that extend beyond the scope of conventional medical management. Instead, these interventions should empower individuals to assume an active role in managing their health. In this context, self-management emerges as a pivotal strategy. Self-management can be defined as the active involvement of individuals in monitoring their health, managing symptoms, adhering to treatments, and making lifestyle changes to maintain well-being [9]. The evidence indicates that effective self-management can mitigate the adverse effects of multimorbidity, delay functional decline, and reduce healthcare utilization [10].

However, the ability to self-manage effectively is not universal and depends on various individual-level factors, including medical health literacy [11]. Health literacy, a multidimensional concept, refers to the capacity to obtain, process, and use health information to make informed decisions [12]. According to the model by Sørensen [13], health literacy contains several domains that each contribute to a person’s health behaviour and their interplay with healthcare services and outcomes. These domains include primarily the ability to first obtain relevant health information, followed by the ability to understand this information, process and interpret the relevant information, and finally apply it to take appropriate action. Due to this multi-dimensionality, in scientific research, multiple definitions and operationalizations of health literacy exist [14], such as a two-tier model describing first the ability to understand and obtain disease-specific information and skills, followed by the ability to interpret and critically assess the obtained information [14, 15]. A recent synthesis of health literacy introduces three dimensions, adding the ability to self-manage health and provider relationships to the previously mentioned knowledge and appraisal dimensions [16]. These working definitions make health literacy an inevitable and highly central ability in healthcare, especially when it comes to the management of multiple illnesses in multimorbid patients. Previous research highlights the central role of health literacy for self-management especially in the face of multimorbidity, as it allows for improved navigation of multiple symptoms, therapy plans and disease courses [11, 17, 18]. Health literacy further directs communication with healthcare providers and steers adherence, all of which impacts not only self-management abilities but also functional health and disease outcomes directly [11]. However, in older adults, health literacy is frequently constrained by cognitive decline, sensory impairments, and limited prior access to education [19]. Despite these challenges, higher health literacy is consistently associated with better chronic disease management, improved medication adherence, and enhanced self-efficacy in navigating complex healthcare systems [20]. Health literacy thus serves as an important and modifiable determinant of health, and understanding how it can be improved specifically in older adults is crucial. Although not all older adults suffer from multiple illnesses simultaneously, the high prevalence of multimorbidity puts health literacy as a central and modifiable determinant of health, and understanding how it can be improved is crucial to aid older adults in their management of multiple health issues and overall age-related decline.

This study investigates the interplay between multimorbidity, health literacy, and functional health in a nationally representative cohort of older adults. Specifically, it examines whether health literacy moderates the relationship between multimorbidity and functional health. Given the increasing prevalence of multimorbidity and its impact on functional health, understanding this relationship is critical for designing interventions that promote successful aging.

## Methods

### Sample

This study utilized data from the “Ageing in Germany (D80+)” survey, a nationally representative sample of individuals aged 80 years and older in Germany, encompassing both community-dwelling and institutionalized participants. Conducted between November 2020 and October 2021 by the University of Cologne in collaboration with the Cologne Center for Ethics, Rights, Economics, and Social Sciences of Health (CERES) and the German Center of Gerontology (DZA), the study was funded by the Federal Ministry for Family Affairs, Senior Citizens, Women, and Youth (BMFSFJ). Study details and data are available at www.dza.de/forschung/fdz/d80/doi/d80-2022-m001. The multi-stage sampling process involved randomly selected municipalities and resident registration data, with samples stratified by gender and age. More methodological details are available in corresponding method papers [21, 22].

The study received ethical approval from the University of Cologne’s Ethics Committee (protocol number: 19–1387_1). Participation was voluntary, with informed consent obtained via questionnaire return or prior to telephone interviews.

Due to the rising prevalence of both multimorbidity and impaired functional health with age [5], and the under-representation of older adults in scientific research [23], understanding how these variables interact is of particular relevance in this age group. We therefore selected all participants for whom the variable of health literacy was available (*n* = 3069).

### Variables of interest

Health Literacy: Health literacy encompasses the ability to access, understand, and use health-related information effectively. As documented in the panel data materials [22], it was assessed using two items focusing on health-related knowledge (”How often do you know what you need to do to stay healthy, get healthy again or strengthen your health” and compliance with this knowledge (“How often do you comply with this knowledge?”). The total score was calculated as the mean of these items, measured on a 4-point Likert scale (1 = very poor, 4 = very good), with higher scores indicating better health literacy.

Multimorbidity: The variable is calculated as a mean score based on the presence or absence of 22 chronic diseases, with a range from 0 to 1 (0 = no conditions, 1 = all conditions present). Higher values indicate greater multimorbidity. The included conditions are: hypertension, diabetes, coronary heart disease, stroke, chronic obstructive pulmonary disease (COPD), asthma, arthritis, osteoporosis, cancer, chronic kidney disease, depression, anxiety disorder, Parkinson’s disease, dementia, hearing impairment, vision impairment, urinary incontinence, chronic pain, obesity, gastrointestinal diseases, liver disease, and other chronic conditions.

Functional Health: Functional health was measured through basal Activities of Daily Living (ADL); this included tasks such as bathing, dressing, and eating. As these most basal but essential daily tasks are often restricted in older adults with multimorbidity [24], due to physical and cognitive impairments, ADLs were used to detect the association between health literacy, multimorbidity and the most crucial daily activities. The variable was calculated as mean scores on 7 items, rated on a 3-point scale from 0 (unable to perform) to 2 (fully independent). The included activities contain eating, dressing, maintaining personal hygiene, walking, rising and laying down, showering/bathing, and going to the toilet. Higher scores represent better functional health [25].

### Covariates

- Age (years), gender (male and female).
- Educational Level: Educational attainment was measured using the International Standard Classification of Education (ISCED) and categorized into three levels: low (primary education), medium (secondary education), and high (tertiary education). This nominal variable reflects socioeconomic background and its potential influence on health outcomes.
- Residential Status: Residential status was categorized nominally, differentiating between private living arrangements and institutionalized care. For heim_gen, the categories were “institutionalized” and “non-institutionalized.” These variables provide insights into environmental factors affecting health outcomes.
- Cognitive Function: Cognitive function was assessed using two components of the DemTect test: semantic word fluency and delayed recall. Semantic word fluency measured the ability to generate words within a category, scored as the raw number of correct words (higher scores indicate better cognitive function). Delayed recall was scored on a scale from 0 to 10, with higher scores representing better memory performance [26].
- Social Network Size: Social connectedness encompasses both loneliness as a subjective feeling as well as social isolation as a more objective measure of social contacts. Both loneliness and social isolation have been shown to strongly impact health and well-being in older age, while loneliness is more closely linked with mental and social isolation with physical health, although both wield strong influences on all aspects of health [27, 28]. Since this analysis is focused on functional health and multimorbidity, social network size was used as a proxy for social isolation, recorded as the number of individuals identified as part of the network [29]. Higher values suggest greater access to social resources, which can act as a protective factor for health [28].
- Depressive symptoms were assessed using the DIA-S4 scale, which provides a summative corrected score for incomplete responses. Higher scores indicate more severe depressive symptoms, with the measure providing insights into mental health status [30].

### Statistical analyses

The statistical analysis was conducted using IBM SPSS Statistics (Version 27) and R (4.1.1). Descriptive statistics were employed to initially characterize the participants. Factors associated with ADL were determined using Elastic Net regression, implemented via the *glmnet* package (4.1–7.1) in R. Elastic Net is a penalized regression technique that combines LASSO (L1) and Ridge (L2) penalties, offering both variable selection and regularization. Predictors were standardized to ensure comparability across variables, and missing values were handled through listwise deletion to maintain consistency in the dataset. A cross-validation approach was utilized to identify the optimal penalty parameter (`lambda`). The parameter alpha = 0.5 was specified to balance the L1 and L2 penalties, providing a mixture of variable selection and shrinkage. The optimal lambda value was identified through 10-fold cross-validation, ensuring a data-driven approach to model regularization. A final Elastic Net model was fitted using this lambda value to identify predictors with non-zero coefficients. These non-zero coefficients were interpreted as the most relevant predictors of basal ADL. To refine the estimates and assess the statistical significance of the predictors, variables retained by the Elastic Net model were included in an Ordinary Least Squares (OLS) regression. This two-step approach allowed for robust predictor identification while enabling hypothesis testing for individual predictors. Multicollinearity was evaluated in the OLS model using Variance Inflation Factors (VIF), ensuring that intercorrelations between predictors did not bias the results.

Moderation analysis was conducted using the PROCESS macro (Version 4.2) in SPSS. This approach facilitated the evaluation of interaction effects between predictors, with a focus on exploring how health literacy moderates the relationship between multimorbidity and ADL.

## Results

### Characteristics of the sample

The sample was composed of individuals with an average age of 86.37 years (SD = 4.35), with a slight predominance of women (51.7%). The mean health literacy score was 3.56 (SD = 0.75) on a scale from 1 to 4, indicating relatively high literacy levels in the sample. The mean score for functional health, as assessed in terms of basal ADL, was 1.76 (SD = 0.41). Mean multimorbidity score was 0.22 (SD = 0.14), with a range of 0.0–0.92. There was considerable variability in educational levels and socioeconomic indicators across the sample **(**Table 1**)**.

### Association between Multimorbidity and functional health

The Elastic Net model revealed a significant negative association between multimorbidity and functional health as measured by ADL performance. For each unit increase in the multimorbidity index, there is a corresponding decrease in functional health of approximately 0.547 points (estimate = −0.547, *p* < 0.0001). Furthermore, age (estimate = −0.0100, *p* < 0.0001) and female gender (estimate = −0.0397, *p* = 0.0130) were found to be associated with a reduction in functional health. The Elastic Net model exhibited moderate explanatory power, as indicated by an R-squared value of 0.14 (F(3,776) = 41.5, *p* < 0.001). The robust association between multimorbidity and functional health persisted even after adjusting for covariates (Table 2).

### Moderating effect of health literacy

The objective of the moderation analysis was to ascertain whether health literacy serves as a moderating factor in the relationship between multimorbidity and functional health. The results indicate a significant interaction between multimorbidity and health literacy, suggesting that the negative impact of multimorbidity on functional health is influenced by the level of health literacy.

The primary effects of multimorbidity and health literacy were both statistically significant. The results demonstrated that multimorbidity negatively predicted ADL (b = −1.6679, SE = 0.4022, *p* < 0.001), which is consistent with the findings from elastic net regularization. The results indicated that health literacy positively predicted functional health (b = 0.0924, SE = 0.0233, *p* = 0.0001), indicating that individuals with higher health literacy tend to have better functional outcomes (R² = 0.15, F(3, 3008) = 110.22, *p* < 0.001).

**Fig. 1:**
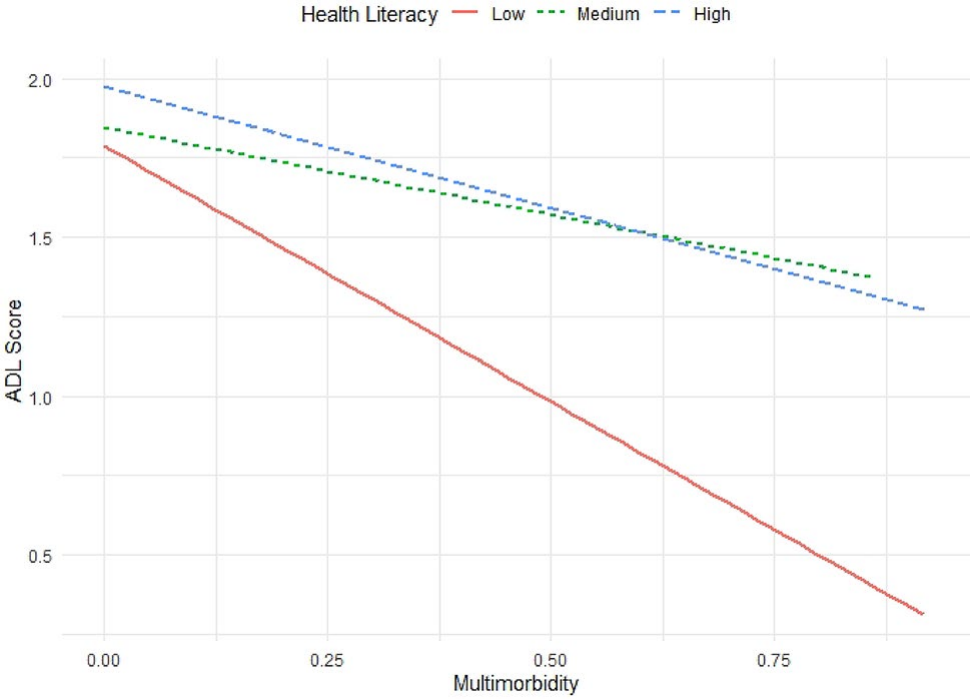
Interaction effect of health literacy and multimorbidity on functional health measured by activities of daily living (ADL)

It is noteworthy that the interaction between multimorbidity and health literacy was statistically significant (b = 0.24, SE = 0.106, *p* = 0.023), indicating that health literacy plays a moderating role in the relationship between multimorbidity and functional health. The conditional effects analysis demonstrated that the negative impact of multimorbidity on functional health was diminished at elevated levels of health literacy. Specifically, at low levels of health literacy (3rd percentile), the effect of multimorbidity on functional health was more pronounced (b = −0.94, SE = 0.09, *p* < 0.001), while at high levels of health literacy (84th percentile), the negative effect was smaller (b = −0.70, SE = 0.06, *p* < 0.001) albeit still present (Fig. 1). As shown in Fig. 1, with increasing multimorbidity, the protective association of high health literacy attenuates and converges with medium health literacy; at higher multimorbidity levels, medium health literacy shows slightly higher predicted functional health than high health literacy.

## Discussion

This study illuminates the complex interrelationship between multimorbidity, health literacy, and functional health, with a particular focus on ADL among older adults. The findings offer valuable insights into the determinants of functional independence and highlight the potential of health literacy as a moderating factor in mitigating the adverse effects of multimorbidity. These results align with and extend existing literature in the field.

This study corroborates the findings of previous research, confirming the substantial impact of multimorbidity on functional health. The presence of multiple chronic conditions was identified as a significant predictor of impaired activities of daily living (ADL), underscoring the cumulative impact of these conditions on an individual’s daily functional health. This finding is consistent with an earlier study that emphasized the bidirectional negative interplay between multimorbidity and functional health over time [7]. This is because the physical capabilities of individuals with chronic conditions such as arthritis, cardiovascular diseases, and diabetes are directly impaired. Furthermore, the indirect effects of these conditions, including increased fatigue, depression, and social withdrawal, compound the challenges associated with maintaining independence [31].

Although multimorbidity has a deleterious impact on functional health, this study highlights the role of health literacy in mitigating this association. Although, in older adults with multiple challenges and impairments, health literacy could not fully buffer the detrimental effect of multimorbidity on functional health, those with higher levels of health literacy demonstrated better functional health despite multimorbidity. The crossing of the medium and high health literacy slopes suggests a non-monotonic moderation: high health literacy does not uniformly buffer loss of functional health at high multimorbidity. One plausible mechanism is ‘over-active’ self-management—patients with very high health literacy may initiate regimen modifications, negotiate or challenge recommendations, or selectively (non-)adhere, which can be counterproductive in complex regimens. Conversely, medium health literacy may foster adherence and reliance on professional guidance. Alternative explanations include measurement artefact (perceived vs. actual health literacy), differential case-mix (greater regimen complexity among high-health literacy patients), and residual confounding by trust/satisfaction, patient activation, or self-efficacy. Hence, health literacy should not be interpreted as a full buffer; its effect likely depends on illness constellation, therapy complexity, and relational factors. Of note, the moderation effect was modest in its magnitude, and in consideration of the simple operationalization of the key constructs with questionnaires and their potential lack of variability in this relatively healthy sample of older adults, these results indicate a context-dependent moderation in an autonomous population of older adults with comparably high health literacy. While the moderation analysis provides first insights into the association between health literacy, multimorbidity and functional impairments, the analyses should be repeated in different populations to arrive at more robust results.

These findings extend those of previous studies conducted with younger adults, which indicated a positive association between the number of physical conditions and difficulties in understanding health information and engaging with healthcare providers [12]. The conditional effects analysis demonstrated that the adverse effect of multimorbidity on functional health was more pronounced among individuals with lower health literacy. This finding highlights the necessity for targeted interventions to enhance health literacy, particularly among vulnerable groups such as those with lower educational attainment or limited access to healthcare resources. This underscores the necessity of adapting health literacy interventions to the particular requirements and circumstances of older adults, acknowledging the heterogeneity in their cognitive, cultural, and socioeconomic backgrounds [19].

In addition to multimorbidity and health literacy, this study identified a number of contextual factors that exert an influence on functional health. Age was identified as a predictor of functional decline, reflecting the physiological changes and increased prevalence of disabilities associated with the aging process. In contrast with the findings of an earlier study, gender differences were observed, with women exhibiting slightly lower functional health scores. This is likely due to a combination of biological, social, and cultural factors [32]. Educational attainment, cognitive function, and social network size were identified as protective factors, thereby reinforcing the multifaceted nature of functional health. These findings are consistent with the conceptual framework proposed by van der Gaag et al. (2022), which emphasizes the interplay between individual, social, and environmental determinants of self-management and functional outcomes [11]. In addition to personal factors such as age and education, contextual and societal factors should be considered when assessing health literacy [33, 34]. The presented data stems from German older adults, a country with excellent health insurance coverage; however, in other countries or in disadvantaged population, system factors such as access to healthcare and health information as well as cultural and economic disparities must be considered. Addressing this topic in disadvantaged groups, such as socially isolated, institutionalized or financially deprived persons, may shed new light on the important dimensions of health literacy and how they translate into the management of multimorbidity and functional impairments.

The findings of this study emphasize the crucial necessity of incorporating health literacy into interventions designed to enhance functional health in older adults both on a personal and organizational level [35]. Furthermore, healthcare providers should adopt a proactive approach to assessing health literacy and addressing any identified gaps through the provision of targeted support. Enhancing health literacy provides a practical and scalable approach to empower individuals to effectively manage their health, particularly in the context of multimorbidity. It is recommended that interventions include tailored educational programs, simplified health communication strategies, and personalized care plans at the individual level. Especially when working with older adults, one step in these interventions includes teaching healthcare providers how to detect gaps in health literacy and how to address them (e.g. by providing understandable communication, using visual aids, allowing space for questions and ensuring the comprehension of information via teach back methods) [36]. According to the Center for Health Care Strategies [37], information materials should further be simplified and tested within the target population, while accessibility to materials must be ensured irrespective of technology use and language. This accessibility encompasses also the utilization of appropriate digital health tools and community-based programs to facilitate the engagement of underserved populations [38].

While this study makes a valuable contribution to the field, it is not without limitations. This cross-sectional analysis precludes causal inference. Likewise, the use of panel-data may introduce bias in the sense that institutionalized or severely ill persons may be excluded from participation, impacting generalizability. The panel-data context may also be the reason for the relatively high mean health literacy, low levels of functional impairments, and low multimorbidity observed in the present sample, which may reduce variability in these key variables. This restriction of range could attenuate observed associations and moderation effects, and the analysis should be repeated in future studies with a more diverse patient population, especially those who are institutionalized or socially excluded. Health literacy was measured by brief self-reports that index perceived rather than actual understanding; older adults may believe they understood instructions yet misapprehend them. Additionally, health literacy is a multi-dimensional construct, of which the instrument utilized in the present analysis only represents two. In future studies, it is crucial to assess the links between the included variables with a more encompassing measurement of health literacy to detect associations between functional health, multimorbidity and different aspects of health literacy. Multimorbidity was operationalized as an unweighted count, ignoring severity, acuity, duration, and management difficulty; different illness constellations are likely unequally manageable. Accordingly, our findings should not be read as health literacy fully buffering multimorbidity—any moderation is partial and context-dependent. Important covariates were unavailable or coarse, including illness type, polypharmacy and regimen/therapy complexity, care coordination burden, motivation, self-efficacy, and trust/satisfaction with providers, leaving residual confounding plausible. Especially social connectedness including social isolation and loneliness should be considered in future research, as they strongly impact health and well-being and constitute a crucial parameter of self-management and health behaviour [27, 28], particularly in older adults with physical or cognitive restrictions who may require the help of other people to manage their health.

## Conclusion

This study provides a first insight into the interplay between health literacy and multimorbidity for functional impairments in older adults. While future studies are required to confirm the outcome in diverse older populations using multidimensional measures of health literacy, its results underscore the significance of addressing both multimorbidity and health literacy in aging populations. As policymakers and healthcare providers endeavor to confront the complexities of multimorbidity, health literacy presents a pragmatic and promising avenue for preserving autonomy and improving health behaviour. It is recommended that future efforts be directed towards the development and implementation of strategies that empower individuals to navigate the complexities of aging and chronic disease management.

**Table 1:** Characteristics of the cohort (*n* = 3069)

		M	SD
Age		85.71	4.15
Health literacy		3.56	0.75
Social Network Size		8.65	6.99
DemTect: semantic word fluency		18.71	6.68
DemTect: Delayed recall		4.59	2.68
Depressive symptoms (DIA-S4)		1.14	1.20
Multimorbidity		0.22	0.14
Functional health: ADL		1.77	0.41

		**N**	**%**
Gender	Male	1562	50.9
	Female	1507	49.1
Level of education	Low	414	13.8
	Medium	1498	50.1
	High	1081	36.1
Full residential care	No	2892	94.7
	Yes	163	5.3
Living situation	Private living	2992	97.5
	Nursing home	77	2.5

**Table 2:** Elastic net regularization: factors associated with functional health

	Estimate	Std. Error	Statistic	*P*-Value
(Intercept)	2.67	0.195	13.7	< 0.001
Age	−0.00816	0.00224	−3.64	0.000
Gender: Female	−0.0463	0.0167	−2.77	0.006
Full residential care: Yes	−0.129	0.118	−1.09	0.277
Nursing Home: Yes	−0.175	0.188	−0.931	0.352
Number of network persons	−0.00107	0.00101	−1.06	0.289
Cognition (Fuency)	0.00236	0.00120	1.96	0.050
Cognition (Recall)	0.00270	0.00310	0.871	0.384
Depressive symptoms	−0.0232	0.00706	−3.29	0.001
Multimorbidity	−0.466	0.0632	−7.37	< 0.001

Note: R² = 0.16, F(9, 770) = 17.0. Variables entered into the model: age, health literacy, gender, education level, full residential care, institutionalized living, social network size, cognition (fluency, recall), depressive symptoms (DIAS4), multimorbidity. Functional health was measured with performance in Activities of Daily Living (ADLs).

## Data Availability

The data used for this manuscript is freely available after registration from [https://www.dza.de/forschung/fdz/d80/doi/d80-2022-m001](https:/www.dza.de/forschung/fdz/d80/doi/d80-2022-m001).
